# Re-thinking osteoarthritis pathogenesis: what can we learn (and what do we need to unlearn) from mouse models about the mechanisms involved in disease development

**DOI:** 10.1186/s13075-023-03042-6

**Published:** 2023-04-12

**Authors:** Raewyn C. Poulsen, Lekha Jain, Nicola Dalbeth

**Affiliations:** 1 Department of Pharmacology & Clinical Pharmacology, Faculty of Medical & Health Sciences, University of Auckland, 85 Park Rd, Grafton, Auckland, 1023 New Zealand; 2 Department of Medicine, Faculty of Medical & Health Sciences, University of Auckland, Auckland, New Zealand

**Keywords:** STR/ORT mouse, Wnt, TGF, CaMKII, Osteoarthritis, Cartilage, Pre-clinical models

## Abstract

Efforts to develop effective disease-modifying drugs to treat osteoarthritis have so far proved unsuccessful with a number of promising drug candidates from pre-clinical studies failing to show efficacy in clinical trials. It is therefore timely to re-evaluate our current understanding of osteoarthritis pathogenesis and the similarities and differences in disease development between commonly used pre-clinical mouse models and human patients. There is substantial heterogeneity between patients presenting with osteoarthritis and mounting evidence that the pathways involved in osteoarthritis (e.g. Wnt signalling) differ between patient sub-groups. There is also emerging evidence that the pathways involved in osteoarthritis differ between the STR/ort mouse model (the most extensively studied mouse model of spontaneously occurring osteoarthritis) and injury-induced osteoarthritis mouse models. For instance, while canonical Wnt signalling is upregulated in the synovium and cartilage at an early stage of disease in injury-induced osteoarthritis mouse models, this does not appear to be the case in the STR/ort mouse. Such findings may prove insightful for understanding the heterogeneity in mechanisms involved in osteoarthritis pathogenesis in human disease. However, it is important to recognise that there are differences between mice and humans in osteoarthritis pathogenesis. A much more extensive array of pathological changes are evident in osteoarthritic joints in individual mice with osteoarthritis compared to individual patients. There are also specified differences in the pathways involved in disease development. For instance, although increased TGF-β signalling is implicated in osteoarthritis development in both mouse models of osteoarthritis and human disease, in mice, this is mainly mediated through TGF-β3 whereas in humans, it is through TGF-β1. Studies in other tissues have shown TGF-β1 is more potent than TGF-β3 in inducing the switch to SMAD1/5 signalling that occurs in osteoarthritic cartilage and that TGF-β1 and TGF-β3 have opposing effects on fibrosis. It is therefore possible that the relative contribution of TGF-β signalling to joint pathology in osteoarthritis differs between murine models and humans. Understanding the similarities and differences in osteoarthritis pathogenesis between mouse models and humans is critical for understanding the translational potential of findings from pre-clinical studies.

## Introduction

Despite decades of research, there are currently no disease-modifying drugs with proven effectiveness for the treatment of osteoarthritis (OA). The extent of failure in translating pre-clinical findings in the OA field means it is time to re-evaluate our understanding of OA pathogenesis and, in particular, to improve our understanding of the similarities and differences in the disease process between pre-clinical mouse models and humans.

There is considerable heterogeneity between patients presenting with OA [1] and mounting evidence that the mechanisms involved in OA differ between different patient subgroups [2, 3]. There are also several different animal models for OA [4-6] with evidence of differences in disease mechanisms between models [7]. This creates an opportunity to learn from these models to further our understanding of inter-individual differences in OA between patients. However, not all findings from animal models align with what is observed in human disease. It is critical that species differences are recognised when extrapolating findings from animal models to human disease to ensure such findings do not become erroneously entrenched in common understanding. The purpose of this review is to compare the disease mechanisms between commonly used preclinical murine models of OA and to highlight where data from these models aligns with observations in human disease and where differences occur.

## Risk factors and causes of OA in humans versus rodent models

OA is a multifactorial disease with both genetic and environmental/lifestyle factors contributing to disease development [8]. Although a number of risk factors for OA have been identified including age, sex, obesity, metabolic syndrome, and prior joint injury [8], why disease develops in some individuals but not others remains incompletely understood.

Many of the known risk factors for OA in humans are also linked with joint pathology in mice, and this has led to the development of different murine models of OA (Table 1). As in humans, age is a risk factor for the development of OA in mice, and some (but not all) mouse strains spontaneously develop OA with age (reviewed in [5]). There is evidence of a potential link between metabolic dysfunction and OA in mice as the STR/ort mouse strain (which is hypercholesterolemic and hyperlipidemic compared to other mouse strains [9]) is particularly susceptible to OA, spontaneously developing OA in multiple joints at a much earlier age than other OA-susceptible mouse strains [10]. Prior joint injury predisposes OA in animals, and it is this finding that has allowed the development of animal models of induced OA. Injury to the joint either by surgical destabilisation or by the injection of substances to degrade joint tissues such as collagenase, results in rapid OA development in mice, rats as well as a number of other animal species [5]. Higher body weight exacerbates OA development following joint injury in mice in line with findings in humans that obesity is a risk factor for OA [8].

**Table 1:** Summary of commonly used murine models of OA

	**Alignment with risk factors for OA in humans**
**Spontaneously occurring OA**	
E.g. aged C57Bl/6	Age
STR/ort mouse	Metabolic syndrome, high body weight
**Injury-induced OA**	
*Surgically induced*	
Destabilised medial meniscus (DMM)	Post-traumatic OA (PTOA)
Anterior cruciate ligament transection (ACLT)	
*Other damage-induced*	
Collagenase induced	Cartilage degradation
Monosodium iodoacetate (MIA), papain	Joint inflammation

There are two major points of difference between mouse models of OA and human disease. One is the effect of mechanical loading differences on joints in small quadruped mice compared to large biped humans. Although this difference is well recognised, to date, it has largely been studied in the context of limb formation during development where the difference in a bipedal versus quadrupedal gait has been shown to dramatically impact cartilage development in the growth plate [11]. The other major difference between mouse models and human disease is the difference in disease susceptibility between the sexes. In mice, there is a profound male bias in OA susceptibility [12, 13] whereas in humans, the female sex is considered a risk factor for OA in joints such as the knee, and there is an increase in the prevalence of OA post-menopause [14]. Ovariectomy increases the susceptibility of female mice to injury-induced OA [12], but initial reports suggest the male bias in OA development in the STR/ort mouse is sex hormone-independent [15, 16].

The vast majority of studies have been conducted using injury-induced models, and therefore, most of our knowledge about the mechanisms involved in OA development comes from studies of injury-induced knee OA in otherwise healthy male mice. Aged mouse models of spontaneously occurring OA have very rarely been studied due to the time and expense involved. The STR/ort mouse is currently the only spontaneously occurring OA mouse model in which detailed studies of the mechanisms involved in OA have been performed.

Although the rapid onset of OA in the STR/ort mouse and injury-induced models makes them ameliorable for study, this also represents a key point of difference between these models and human disease. The rapid onset of OA in these murine models may indicate that the magnitude of disease-causing pathway activity and/or the array of different disease-inducing pathways involved differs from that in humans. In this review, we compare the findings from injury-induced OA models and the STR/ort mouse to those in human disease.

## Histological appearance of OA in human disease vs mouse models

In humans, OA is associated with pathological changes in multiple joint tissues including the cartilage, subchondral bone, synovium, ligaments, and meniscus as well as a change in the composition of the fat pad [17-22]. These changes lead to structural joint remodelling resulting in impaired joint mobility and pain. As the cartilage, bone, and synovium have been the most well-studied, these tissues are the focus of this review.

### Cartilage

#### Human disease

Both the amount and the composition of articular cartilage change in OA. During early OA, the water content of cartilage increases in conjunction with changes in proteoglycan content, and the resultant cartilage swelling can result in a temporary increase in cartilage thickness [23-25]. Continual proteoglycan loss and type II collagen degradation lead to net cartilage attrition, and this is a major cause of joint space narrowing in disease [25]. Cartilage calcification is common (reviewed in [26]) with deposits of calcium crystals such as basic calcium phosphate (BCP) and calcium pyrophosphate dihydrate (CPP) frequently found in cartilage as well as other joint tissues in OA [26]. These changes in the cartilage are accompanied by altered activity of chondrocytes within the affected tissue. Normally, chondrocytes in healthy adult cartilage exist in a state of replicative quiescence [27]. However, a shift in chondrocyte phenotype occurs in OA, and proliferative, dedifferentiated, hypertrophic, senescent, and apoptotic chondrocytes have been detected in OA cartilage [27]. Chondrocyte catabolic activity increases substantially [28-30], and chondrocytes display altered synthetic activity producing structurally inferior cartilage with an abnormal collagen and proteoglycan content (reviewed in [30]). This contributes to cartilage loss [30]. In humans, there is evidence of inter-individual variability in the changes in chondrocyte phenotype that occur in OA. For instance, terminally differentiated hypertrophic chondrocytes have been detected in some but not all patients [31], and there are marked differences in the level of chondrocyte apoptosis reported in human OA with estimates ranging from 6 to 88% of the total cell population [32, 33].

#### Mouse models

Cartilage loss is also a key feature of OA in mouse models. However, the non-calcified cartilage layer in mouse joints is approximately 50-fold thinner than in humans [34], and OA induction post-injury often leads to rapid full-thickness cartilage loss in these animals [35]. Although the speed of cartilage loss in mice post-injury varies between models depending on the degree of joint instability [36], cartilage loss is still considerably more rapid than in humans where full-thickness cartilage loss is usually only apparent at late-stage disease [35]. Whether cartilage loss is preceded by cartilage swelling in mouse models of OA is unclear.

There are similarities in the changes in chondrocyte phenotype observed in mouse models compared to human OA [10, 37-39]. For instance, in collagenase-induced and surgical injury-induced OA models as well as the STR/ort mouse, marked increases in chondrocyte apoptosis have consistently been observed [38-40]. Chondrocytes expressing hypertrophy markers such as type X collagen have also consistently been detected in injury-induced OA models from an early stage of OA development, and chondrocyte hypertrophy is implicated as a cause of cartilage loss in these models [36, 41]. Although fewer studies have been conducted with the STR/ort mouse, the number of type X collagen-positive cells has also been found to be markedly elevated in these animals [42]. Therefore, although there may be heterogeneity amongst patients in the degree of chondrocyte apoptosis and hypertrophy in OA, these appear to be a common feature of OA in mouse models [14].

### Synovium

#### Human disease

In OA, synovitis is associated with fibrosis, increased vascularity, hyperplasia, and inflammation [43, 44]. There is an increase in the proliferation of the resident fibroblast-like synoviocytes (FLS), and immune cell infiltration can occur with macrophage, T cell, and mast cell enrichment evident [45, 46]. Synovitis is a feature of OA in some but not all patients and can vary in location, severity, and potentially with disease stage [47, 48]. The site at which synovitis occurs may influence symptom manifestation [49]. For instance, suprapatellar synovitis has been found to be strongly correlated with suprapatellar pain [50].

Estimates of the prevalence of synovitis in human OA vary. For instance, one study found synovitis was present in approximately 67% of patients at the time of joint replacement surgery [51] whereas another found synovitis in 89.2% of patients [52]. In comparison, only 8.4–10.3% of individuals with no radiographic evidence of OA were found to show signs of synovitis [53]. Synovitis is linked with both increased risk of OA and increased severity of disease [54, 55].

#### Mouse models

In contrast to humans, synovitis appears to be a ubiquitous feature of the disease in injury-induced murine OA models, and there is emerging evidence that this may also be the case in the STR/ort mouse [10, 56]. Changes in the synovium are evident prior to cartilage loss in the DMM model [56] and synovitis has been shown to contribute to cartilage loss and pain in surgically induced and collagenase-induced OA models [57]. Increased FLS proliferation as well as increased inflammatory cytokine levels are evident in the synovium of STR/ort mice [58]. However, in contrast to human disease, the STR/ort mouse also shows increased systemic inflammation [58] and OA develops in multiple joints in this animal [10, 59].

### Bone

#### Human disease

Changes in both the cortical subchondral bone plate and underlying trabecular bone are evident in patients with OA [60-62]. In humans, subchondral bone turnover is increased, with increased formation and activity of osteoblasts and osteoclasts and altered osteocyte signalling (reviewed in [63]). This leads to initial subchondral bone thinning, but in late-stage disease, subchondral bone thickness is increased, and bone sclerosis is common [64]. Osteophytes are also commonly associated with OA; however, they are not a specific feature of OA in humans. Rather, the number and/or size of osteophytes is often increased in OA [65, 66]. One study reported 98.1% of patients with knee OA had radiographic evidence of osteophytes [65]. In comparison, osteophytes were detected in 54.3% of individuals in a similar-aged general population and in 69.6% of individuals aged less than 30 years indicating they are also highly prevalent in non-OA joints [66]. Other structural abnormalities such as bone cysts and bone marrow lesions (BMLs) are present in some but not all patients with a prevalence of 66% and 30.6%, respectively, reported in patients with knee OA [65, 67]. Both BMLs and cysts have been associated with increased cartilage degradation and hence their presence in a joint may lead to more severe OA [68]. Although it remains unclear whether changes in subchondral bone precede changes in the cartilage [19], it is clear that changes in bone occur at a very early stage of disease in humans.

#### Mouse models

Similar to humans, initial subchondral bone thinning followed by subsequent thickening and sclerosis has also been observed in injury-induced murine OA models and both osteoblast and osteoclast activity is elevated [69]. Although changes in subchondral bone have been less extensively studied in the STR/ort mouse, these mice naturally display a high bone mass phenotype, developing both bone sclerosis and osteophytes [70]. However, although bone marrow-derived osteoblast activity was found to be higher in STR/ort mice, bone marrow-derived osteoclasts showed markedly lower resorptive activity [70]. This is in direct contrast to injury-induced models and humans and suggests that bone formation is uncoupled from bone resorption in the STR/ort mouse and that bone formation alone rather than bone turnover is upregulated in this model.

Unlike in humans, osteophytes are often either not detected or present at only minimal levels in non-OA joints in mice and the development of osteophytes appears to be a specific feature of OA [36]. The site of osteophyte formation differs depending on the method used for surgical OA induction [36] suggesting osteophyte formation is a direct consequence of joint injury in these models. The small size of mouse joints hinders the detection of BMLs and cysts; however, BML formation has been detected following ACLT in mice using micro-MRI (magnetic resonance imaging) [71]. In larger animal models such as rats, BMLs and cysts have been detected in up to 100% of affected joints following surgical OA induction [72, 73]. BMLs and cysts may therefore be a more ubiquitous feature of OA in animal models than they are in human patients.

**Fig. 1:**
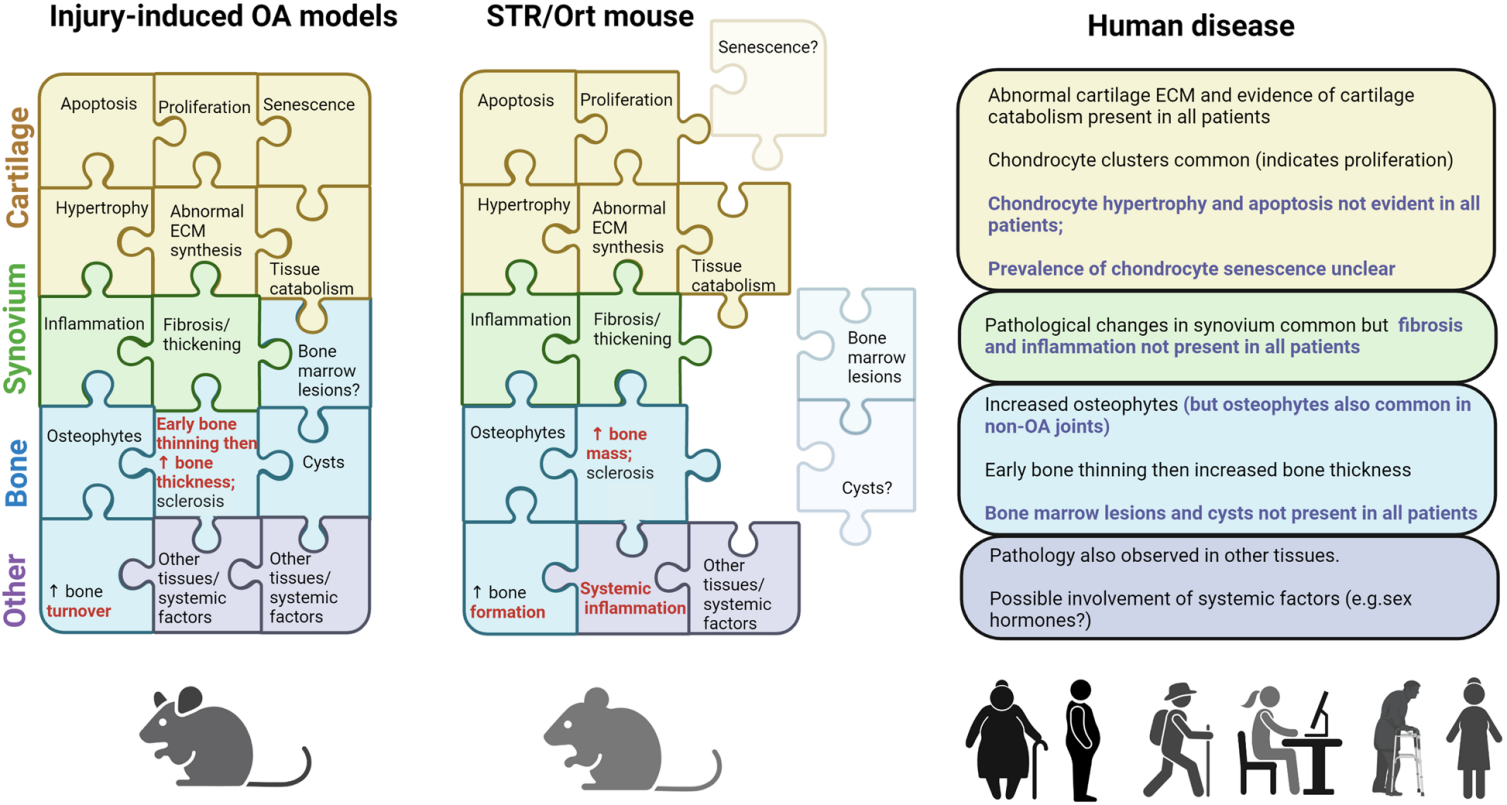
Histological changes in the cartilage, synovium, and bone observed in injury-induced mouse models of OA, the STR/ort mouse, and human patients. A range of pathological changes occurs in joint tissues during disease development in mouse models of OA. While these changes are also evident in human disease, there is heterogeneity between patients in the array of changes present and only a subset, rather than the full spectrum of pathological changes may be present in any one patient with OA. These differences are highlighted in blue text. In addition to cartilage, bone, and synovium, pathological changes in other joint tissues such as the ligaments, meniscus, and fat pad as well as systemic factors such as hormone levels, inflammation also contribute to disease. Differences in histological observations between injury-induced mouse models and the STR/ort mouse are highlighted in red text. The figure was created using Biorender.com

Overall, although the structural changes that occur in OA in mouse models are similar to those that have been described in populations of OA patients, individual mice display a more extensive array of such changes than individual patients. Figure 1 summarises the histological changes seen in OA in injury-induced models, the STR/ort mouse and human patients.

Similarities and differences in the structural changes in joint tissues between mouse models and human disease imply that there are similarities and differences in the mechanisms driving OA pathology between the two. There is evidence that this is the case. Although a number of different pathways contribute to OA development, here we review the Wnt (wingless-type) and TGF (transforming growth factor) pathways, two major signalling cascades implicated in OA pathology in multiple joint tissues.

## Wnt signalling in OA: human disease vs. mouse models

### Overview of the Wnt signalling pathway

**Fig. 2:**
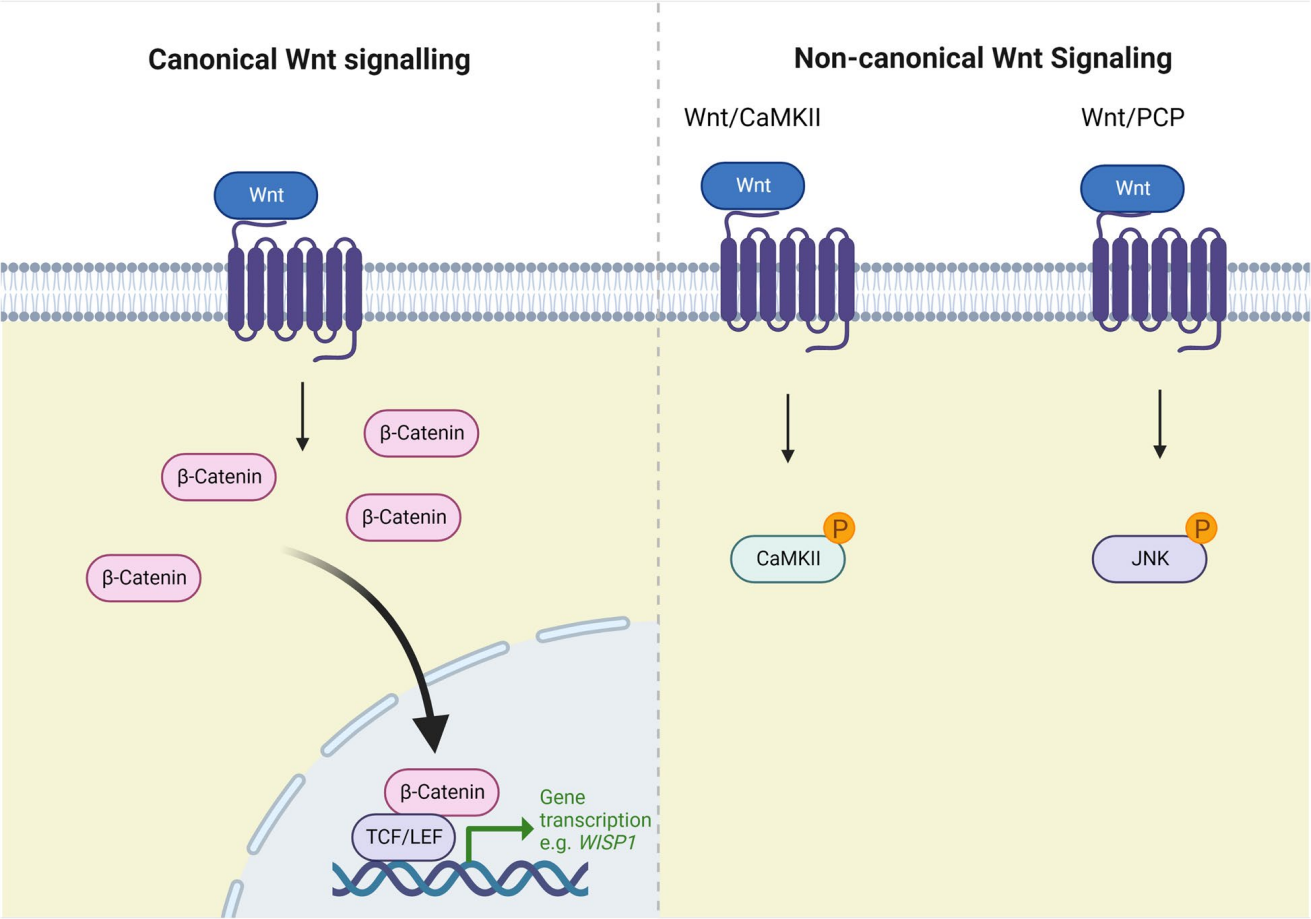
Simplified schematic of the Wnt signalling pathway. Wnt signalling occurs through canonical and non-canonical pathways. Two of the major non-canonical pathways are Wnt/CaMKII and Wnt/PCP. Members of the Wnt ligand family can bind to a multitude of different receptors. Different Wnt ligand/receptor combinations lead to differences in the relative amount of canonical and non-canonical pathway activation. The figure was created using Biorender.com

Wnt signalling is critical for the regulation of cell differentiation and tissue development [74]. In musculoskeletal tissues, Wnt signalling is activated by mechanical injury [75] and controlled by a variety of different growth factors including TGF-β [76]. Wnt signalling occurs by both canonical and non-canonical pathways, each of which results in different outcomes on cell activity. Canonical Wnt signalling is mediated through the transcriptional regulator β-catenin leading to altered expression of β-catenin target genes. In contrast, non-canonical Wnt signalling is β-catenin independent and involves the activation of specific intracellular kinase cascades. There are several different non-canonical Wnt pathways, but two of the major pathways are the planar cell polarity (PCP) pathway mediated through the activation of Jun kinase (JNK) and the Ca^2+^/CaMKII (calcium/calmodulin kinase II) pathway [77] (Fig. 2).

Wnt signalling is initiated by Wnt ligand/receptor binding. There are 19 different Wnt ligands as well as a multitude of different Wnt receptors including those of the frizzled (FZD) family as well as others, e.g. RYK (receptor tyrosine kinase related) and ROR1/2 (RAR related orphan receptor) [77]. Wnt ligands and receptors are expressed by a wide variety of different tissues including the cartilage, bone, and synovium [78, 79]. Although traditionally Wnt ligands were classified as either “canonical” or “non-canonical” based on their purported preference for Wnt pathway activation, it is now known that the same Wnt ligand can have different effects when acting on different receptors [77], and the cellular context influences the outcome of WNT signalling [74]. This means that the same Wnt ligand can have different effects on different cells or tissues.

### Role of Wnt signalling in normal joint biology

The effects of canonical Wnt signalling on joint biology and OA development have been extensively studied (reviewed in [80]). Less is known about the effects of non-canonical Wnt signalling. To date, the Wnt/CaMKII pathway has received the most research attention [81-83]. Currently, very little is known about the role of pathways such as Wnt/PCP although one study has shown that activation of Wnt/PCP protects against cartilage loss in mice suggesting it may have a cartilage-protective role [84].

Both canonical and non-canonical Wnt/CaMKII signalling regulate chondrocyte and osteoblast differentiation [85-89]. Both also influence the activity of cells in the synovium. For instance, increased canonical Wnt signalling in the synovium has been shown to lead to increased cartilage-degrading enzyme production [90], whereas elevated non-canonical Wnt/CaMKII activity has been linked with synovial inflammation [91]. The effects of both the canonical pathway and non-canonical Wnt/CaMKII signalling may depend on the level of pathway activation. Low-level canonical Wnt signalling aids in maintaining the normal partially differentiated state of adult chondrocytes whereas excessive Wnt signalling promotes chondrocyte terminal differentiation and hypertrophy [86]. Similarly, both excessive activation and inhibition of CaMKII signalling have been shown to result in cartilage loss [82, 87, 88].

## Changes in Wnt signalling in OA: animal models vs. human disease

### Cartilage

#### Human disease

Both β-catenin and phosphorylated CaMKII have been found to be elevated in human OA cartilage [81, 92]. However, whether both are upregulated at the same time in the same patients or whether there is heterogeneity in canonical versus non-canonical Wnt signalling between patients remains unclear. In potential support of the latter, a large-scale RNA-seq study of human OA cartilage found patients could be stratified into two groups with differential levels of expression of genes linked to Wnt signalling a key discriminator between the two [2]. Although Wnt16, a ligand shown to activate Wnt/PCP signalling in cartilage in mice [84], has been shown to be upregulated in human OA cartilage [84], it is unclear whether the profile of Wnt receptors is the same in murine compared to human tissue and whether Wnt ligands have a similar effect in both. It therefore remains to be determined whether Wnt/PCP signalling is also elevated in human OA cartilage. One of the difficulties in assessing non-canonical Wnt activity in human tissue is that kinases such as CaMKII and JNK can also be activated by pathways other than Wnt [93, 94]. Determining whether Wnt signalling is a likely cause of kinase activation requires multiple levels of evidence.

#### Mouse models

Increased canonical Wnt activity has been observed in the cartilage in injury-induced OA models as well as in the STR/ort mouse; however, the time at which this occurs during disease development appears to differ between the two. In the collagenase-induced mouse model, mRNA levels of *wisp1* (WNT1-inducible signalling pathway protein 1, a Wnt1 and β-catenin inducible gene) were increased as early as 1 week post-collagenase injection and β-catenin levels increased at 3 weeks indicating rapid activation of canonical Wnt signalling in cartilage [7]. Similarly, microarray data indicates that Wnt signalling is also upregulated from an early stage of disease in the DMM model [95]. In contrast, increased Wnt ligand and *wisp1* expression were only observed in the cartilage of the STR/ort mouse at a late-stage disease when substantial cartilage damage was already apparent [7]. This difference in timing is relevant as Wnt signalling is known to be activated in response to tissue damage [96]. These findings may imply that canonical Wnt signalling has a causal role in early cartilage loss in injury-induced models but may only be upregulated in chondrocytes in the STR/ort mouse as a consequence of disease-induced cartilage damage. Although this likely exacerbates cartilage loss at a late-stage disease in the STR/ort mouse, the overall contribution of Wnt signalling to cartilage loss may be lower in this model than in injury-induced models.

Both the non-canonical Wnt/CaMKII and Wnt/PCP pathways have been found to be upregulated in injury-induced OA mouse models and both have been shown to limit cartilage loss in these models [81, 97]. As non-canonical Wnt pathways inhibit the canonical pathway, this may be a consequence of the dampening of canonical Wnt signalling activity [88, 98]. Whether these non-canonical pathways are also activated in the STR/ort mouse is unclear.

An important consideration when comparing studies in murine models to those in humans is that the small size of mouse joints often necessitates the use of different experimental approaches than those used in humans. This is particularly of consequence for studies at the transcriptomic or proteomic level which are frequently used to screen for signalling pathway differences in disease. In murine cartilage, often the entire femoral condyle (as opposed to just articular cartilage) is used for analysis [99]. However, in mice, the growth plate does not ossify [90] meaning that both growth plate cartilage and articular cartilage may be present in samples [99]. Damage to both articular and growth plate cartilage has been shown to occur in injury-induced OA models [100]. Although growth plate damage may not contribute to articular cartilage loss in these models [100], it could feasibly alter signalling pathway activity (particularly damage-responsive pathways such as Wnt) in chondrocytes within the growth plate. This may confound data interpretation. Currently, many studies in mouse models have relied on transcriptomic data to determine Wnt signalling activity in the cartilage. It is possible that some of the variation in Wnt signalling seen between models and/or between these models and human disease is caused by methodological differences.

### Synovium

#### Human disease

There is a paucity of data from human studies examining Wnt signalling in the synovium in OA, and mixed results have been reported. A modest increase in mRNA levels of *AXIN2* (axis inhibition protein 2), a regulator of β-catenin stability, was detected in OA synovial fibroblasts in one study [101], and mRNA levels of *WISP1* were found to be elevated in the synovium from OA patients in another [7] suggesting increased canonical Wnt activity. However, more recently, the overall level and nuclear localisation of β-catenin were found to be lower whereas levels of phosphorylated CaMKII and components of the Wnt/PCP pathways were found to be elevated in synovial fibroblasts from patients with OA compared to age-matched healthy controls, indicating increased non-canonical rather than canonical Wnt signalling in human OA synovium [102].

#### Mouse models

In contrast to humans, there is substantial evidence that canonical Wnt signalling is upregulated in the synovium in both injury-induced mouse models as well as the STR/ort mouse; however, again, there is a difference in the timing at which this occurs during disease development. In the collagenase-induced OA mouse model, both β-catenin and *wisp1* levels were found to be elevated in the synovium at early stages of disease; however, although Wnt ligand levels were upregulated early in disease in the STR/ort mouse, *wisp1* levels were again only elevated at late-stage disease [7]. Given the finding that Wnt ligand levels were upregulated in the STR/ort mouse early in the disease but no change in canonical Wnt signalling was evident at this point, it would be interesting to determine whether non-canonical pathways are activated at early disease stages in this model. Currently, it is unclear whether Wnt/CaMKII or Wnt/PCP signalling is upregulated in the synovium in mouse models of OA.

### Bone

#### Human disease

Canonical Wnt pathway activity has been shown to be downregulated in osteoblasts and bone marrow mesenchymal stem cells from patients with OA [103]. Conversely, osteoblasts from OA subchondral bone have been shown to express higher levels of genes associated with non-canonical Wnt pathways such as Wnt/PCP [104] suggesting non-canonical rather than canonical Wnt signalling is activated in human OA subchondral bone.

#### Mouse models

In contrast to human disease, canonical Wnt signalling activity has been found to be elevated in the subchondral bone in injury-induced OA mouse models although this increase occurs at a relatively late stage of disease in these animals [105]. To our knowledge, Wnt signalling has not yet been examined within the bone in the STR/ort mouse. It would be interesting to determine if, as for cartilage and synovium, Wnt signalling activation differs between the STR/ort mouse and injury-induced models.

**Fig. 3:**
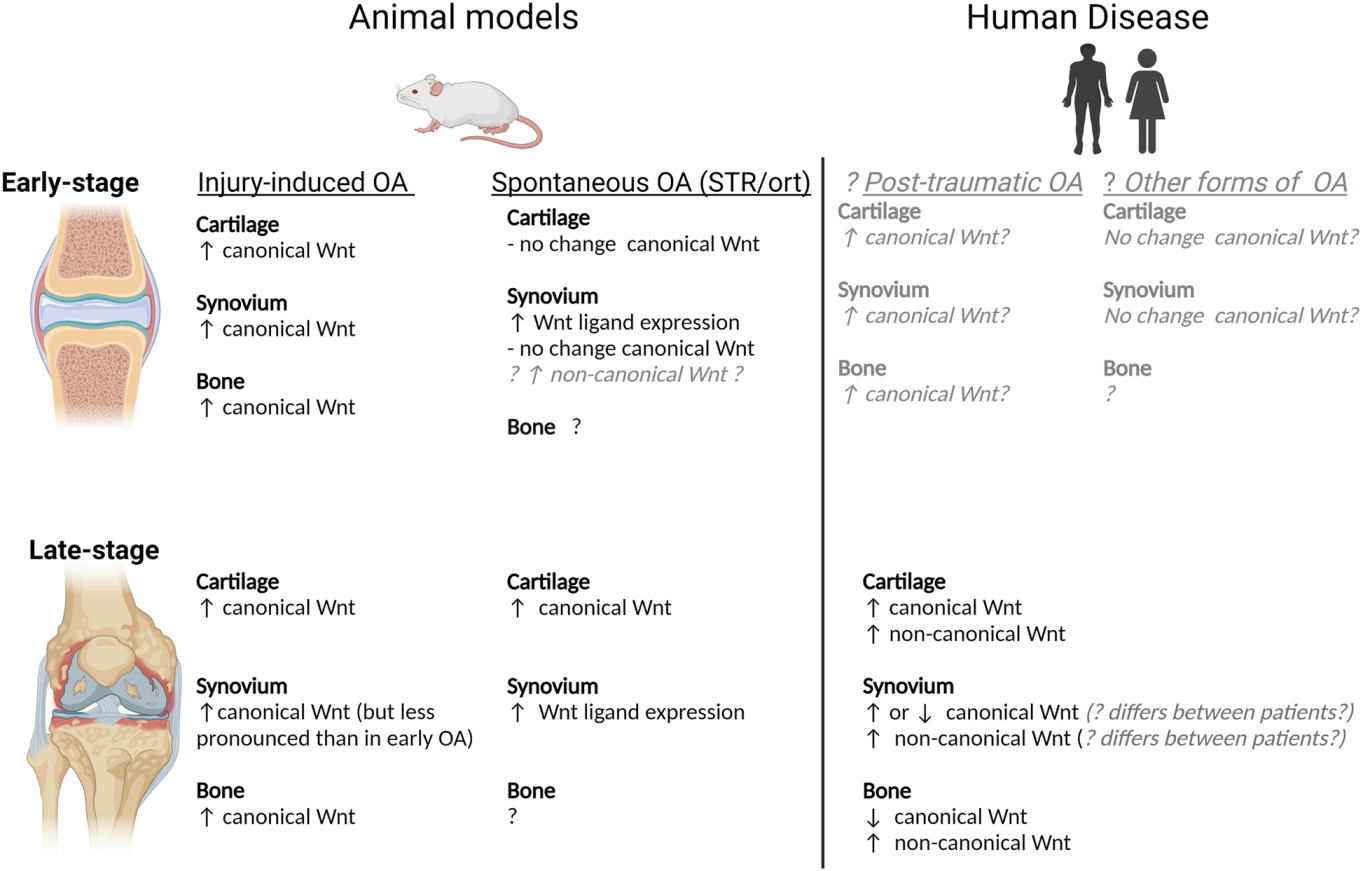
Comparison of the changes in Wnt signalling pathway activity observed in animal models of OA compared to human disease. Observations regarding Wnt signalling activity in human disease have been largely limited to late-stage disease. Findings from animal models may aid in predicting the changes in Wnt signalling that occur during early-stage disease in humans (grey text). Differences in Wnt signalling between injury-induced animal models of OA and the STR/ort mouse may suggest that Wnt signalling also differs between patients with post-traumatic OA compared to other forms of OA. The figure was created using Biorender.com

Taken together, findings from animal models indicate that canonical Wnt signalling is upregulated at a much earlier stage of disease in both cartilage and synovium in injury-induced models compared to the STR/ort mouse. Pharmacological inhibition of canonical Wnt signalling or its downstream effectors has consistently been shown to protect against cartilage loss in injury-induced mouse models [103]  demonstrating that increased canonical Wnt signalling has a major contribution to OA development in these models. To our knowledge, the effect of canonical Wnt inhibitors has yet to be examined in the STR/ort mouse, but it would be interesting to determine whether canonical Wnt inhibition is less effective at preventing cartilage loss in this model than in injury-induced models. This is particularly relevant given emerging evidence that there is heterogeneity in Wnt signalling between patients with OA and in light of recent findings that the canonical Wnt inhibitor lorecivivint showed efficacy in reducing joint space narrowing in only a small subgroup of patients [106]. Considering observations from animal models, it is possible that canonical Wnt signalling differs between patients with post-traumatic OA and those with other forms of OA and this may account for some of the heterogeneity in Wnt signalling observed between patients (Fig. 3).

## TGF signalling in OA: human disease vs. mouse models

### Overview of the TGF signalling pathway

Like WNT signalling, TGF signalling has a fundamental role in regulating musculoskeletal cell differentiation. Both osteoblasts and chondrocytes produce a range of TGF-β superfamily ligands. These are secreted as inactive pro-proteins which become embedded in the extracellular matrix of bone and cartilage [107, 108]. Tissue breakdown (for instance due to osteoclast activity and/or mechanical loading) initiates the release and cleavage of these ligands leading to the initiation of TGF/BMP signalling [107, 108].

**Fig. 4:**
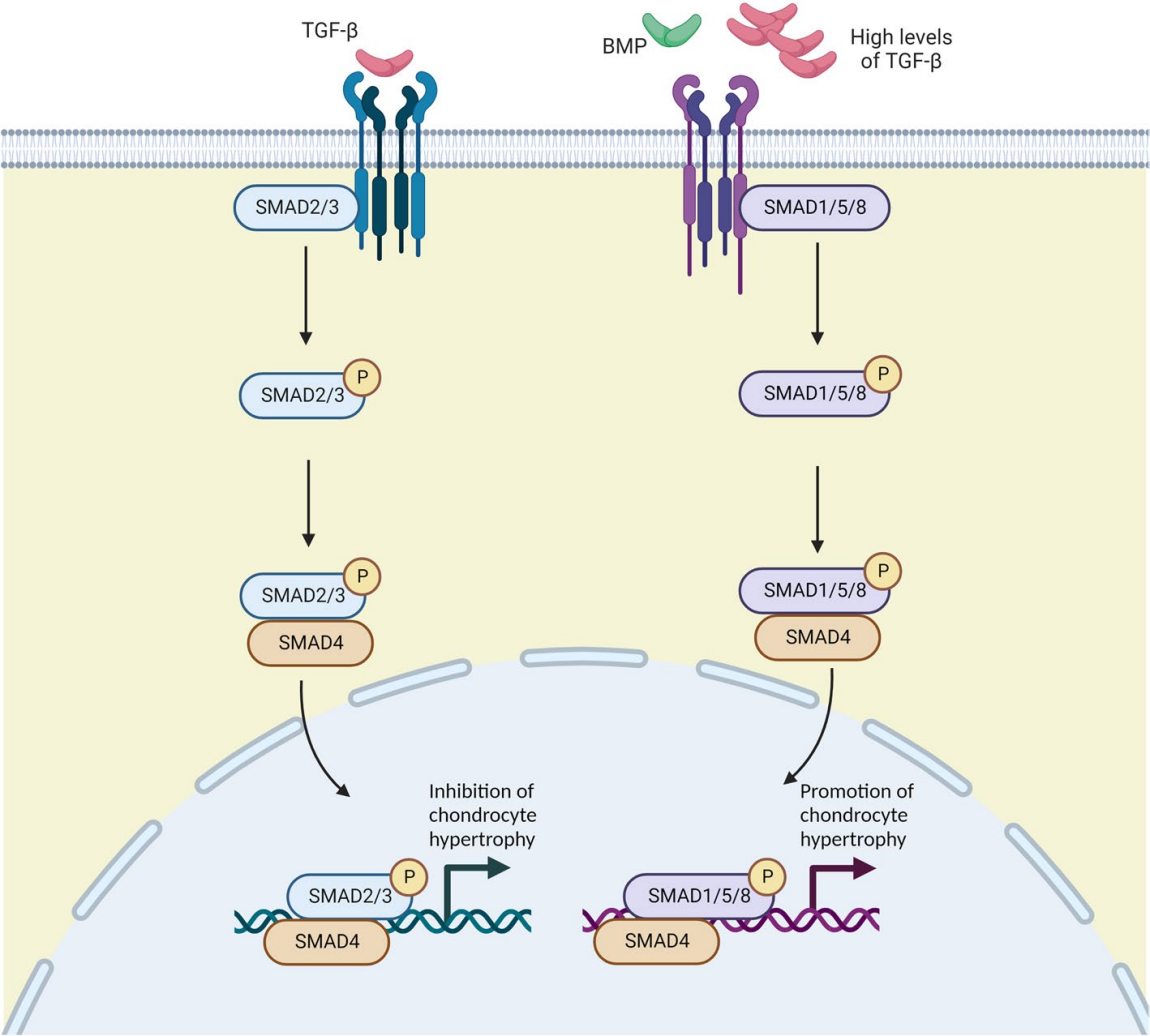
Simplified schematic of the TGF/BMP signalling pathway. TGF-β and BMP family ligands typically signal through different receptors. This results in differences in SMAD activation with TGF-β typically activating SMAD2/3 signalling and BMP typically activating SMAD1/5/8 signalling. Activation and SMAD-mediated gene transcription. However, at high concentrations, TGF-β can also induce SMAD1/5/8 signalling. In chondrocytes, this leads to stimulation rather than inhibition of chondrocyte hypertrophy. The figure was created using Biorender.com

One of the major mechanisms through which TGF signalling occurs is via the activation of the R-SMAD family of transcriptional regulators. Different TGF ligands (which include those of the TGF-β, bone morphogenic protein (BMP), and growth and differentiation factor (GDF) families [109]) preferentially activate different TGF receptors leading to differences in the profile of R-SMADS activated. For instance, BMP-mediated signalling leads to SMAD1/5 activation whereas TGF-β-mediated signalling leads to SMAD2/3 activation. However, at high concentrations, TGF-β ligands switch their receptor binding preference leading to SMAD1/5 activation rather than SMAD2/3 [110-112]. This has important consequences particularly for chondrocytes as the two pathways have opposing effects on chondrocyte differentiation. Whereas SMAD2/3 signalling suppresses chondrocyte terminal differentiation and hypertrophy, SMAD 1/5 signalling promotes it [113, 114] (Fig. 4).

## Changes in TGF signalling in OA: mouse models vs human disease

### Cartilage

#### Human disease

TGF-β levels in cartilage are reduced in OA [115], and this is believed to be a major contributor to the increase in TGF-β concentration in synovial fluid observed in patients with OA [116]. TGF-β release from the cartilage therefore has can lead to an overall increase in TGF-β-mediated signalling in all joint tissues exposed to synovial fluid. In OA chondrocytes, increased levels of free TGF-β contribute to a shift in the balance of SMAD activation in OA chondrocytes such that SMAD 1/5 signalling dominates at the expense of SMAD 2/3 [117].

#### Mouse models

**Fig. 5:**
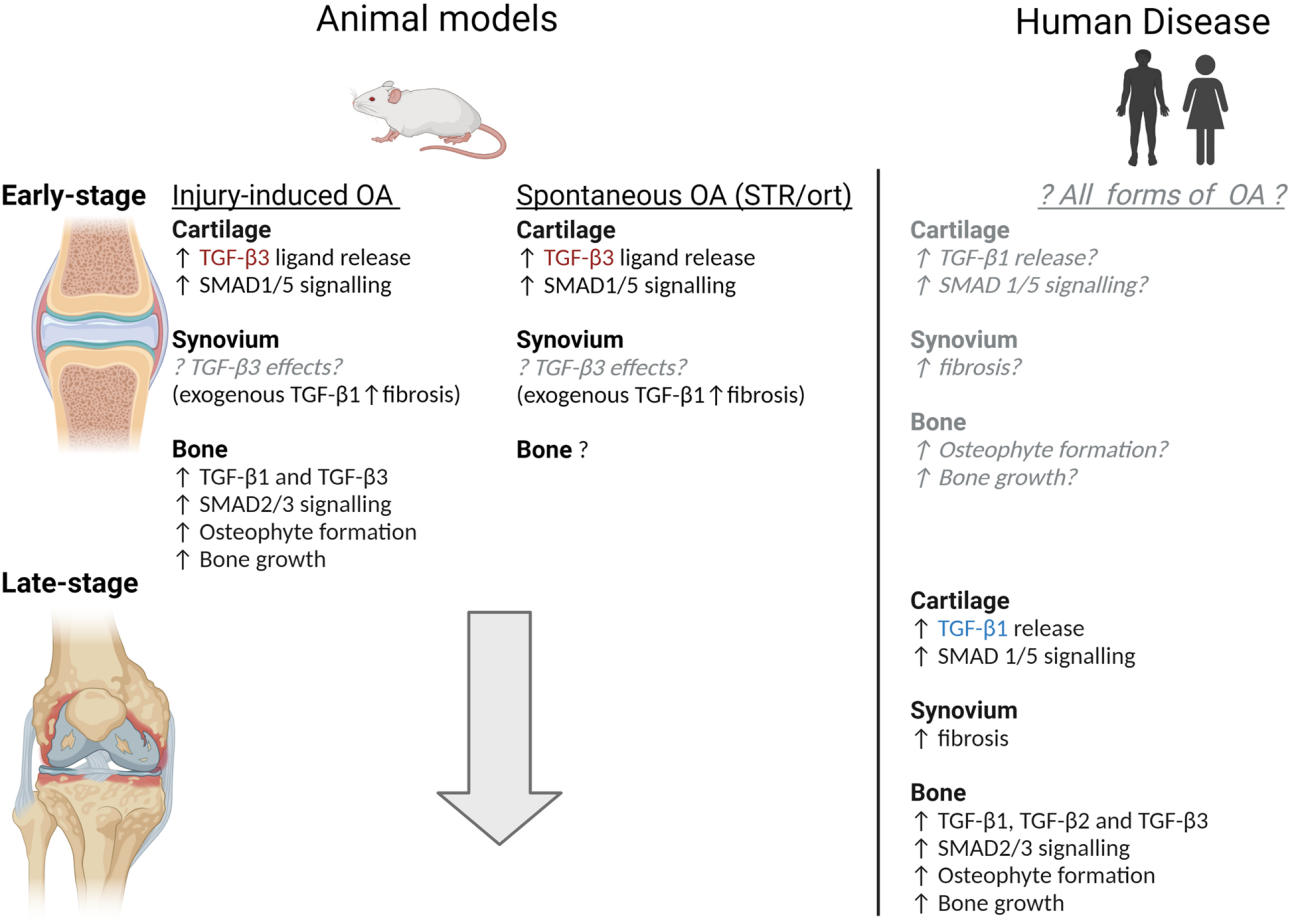
Comparison of TGF signalling in the cartilage, synovium, and bone in animal models of OA compared to human disease. Observations regarding TGF signalling activity in human disease have been limited to late-stage disease. Findings from animal models may aid in predicting the changes in TGF signalling that occur during early-stage disease in humans (grey text). One key point of difference in TGF signalling in mouse models compared to humans is that in mice, TGF-β3 is the most abundant TGF-β isoform in cartilage and the major form of TGF-β released in OA whereas in humans it is TGF-β1 (highlighted in red text). TGF-β1 and TGF-β3 have been shown to have different effects in other tissues. Whether they also have different effects on joint tissues in OA remains to be determined. The figure was created using Biorender.com

Decreased TGF-β levels in the cartilage have been observed in both the STR/ort mouse and the collagenase-induced OA model, consistent with increased TGF-β release [118]. In both models, these changes occurred at early disease stages [118] (Fig. 5).

However, unlike in humans where TGF-β1 is the major TGF ligand present in cartilage and the dominant TGF-β ligand involved in OA [115], in murine models, it is TGF-β3 [13]. This is potentially relevant because TGF-β1 has been found to be more potent than TGF-β3, inducing SMAD1/5 activation at a lower concentration than TGF-β3 in bovine endothelial cells [112]. Whether this also occurs in chondrocytes is unknown. However, this may mean that the pathological switch to SMAD1/5 signalling occurs at a lower TGF-β concentration in human disease compared to murine models.

### Synovium

#### Human disease

Synovial fibroblasts are also responsive to increased TGF-β levels and expression of TGF-β target genes has been shown to be elevated in the synovium from patients with OA [119].

#### Mouse models

Expression of TGF-β responsive genes has also been found to be upregulated in the synovium of the collagenase-induced mouse although the panel of genes upregulated in this model differed from that observed in human disease [119]. TGF-β1 has also been shown to promote synovial fibrosis in mice [119]. However, to our knowledge, the effects of TGF-β3 on synovial fibrosis have not been examined. This would be relevant to determine as in skin the two TGF-β isoforms have opposing effects on fibrosis; TGF-β1 promotes scar formation and fibrosis whereas TGF-β3 inhibits it [120]. It is therefore possible that the difference in TGF-β isoform expression in mice versus humans means that TGF-β signalling has a greater role in promoting synovial fibrosis in human disease than it does in murine models of OA.

### Bone

#### Human disease

Expression of TGF-β1, TGF-β2, and TGF-β3 has been found to be increased in osteoblasts in subchondral bone from patients with OA [72]; however, our knowledge regarding the consequences of increased TGF ligand levels in OA bone largely comes from observations from murine models.

#### Mouse models

TGF-β1, TGF-β3, and phosphorylated SMAD2 have been shown to be elevated at locations where osteophyte formation is common in both injury-induced mouse models, and the STR/ort mouse [121] and increased TGF-β signalling has been shown to lead to increased bone growth and osteophyte formation [71].

That similar TGF-β ligands appear to be upregulated in the bone in both mouse models of OA, and human disease suggests the effect of TGF-β signalling on bone growth and osteophyte formation may be similar between the two. However, the effects of TGF signalling may differ in the synovium and cartilage between mouse models and human disease due to the differences in the TGF-β isoforms involved (Fig. 5). Given there is now considerable evidence that TGF-β1 and TGF-β3 have differing effects in other cell types, it is important that the implications of the difference in TGF-β isoform involvement in the cartilage and synovium are re-evaluated.

## Conclusions and future direction

### What can we learn from mouse models?

Although fewer studies have been conducted with the STR/ort mouse compared to injury-induced OA models, there is strong evidence that the mechanisms involved in OA differ between this model and injury-induced models. Understanding the causes and consequences of the mechanistic difference between these models may prove valuable for understanding inter-individual differences in human disease particularly between patients with post-traumatic OA and other forms of OA. Although studies in aged mouse models of spontaneously occurring OA are much more time-consuming and expensive than other models, comparing disease mechanisms in these animals to those in the STR/ort mouse is also likely to be valuable and potentially contribute to our understanding of the mechanisms involved in metabolic OA.

### What do we need to unlearn from mouse models?

Development of OA is much more rapid in mouse models than in humans. The rapid onset of OA in injury-induced models is consistent with a greater range of pathological mechanisms acting from an earlier disease stage in these models than in humans. It is critical that this is recognised. Although it is common for studies to provide confirmatory data from human tissue when findings from mouse models align with human disease, very few studies highlight when data does not align but this is equally as important. No animal model completely recapitulates human disease. This is particularly the case with murine pre-clinical models where the profound male bias in OA susceptibility observed in these models is clearly not recapitulated in humans and differences in joint loading due to quadrupedal versus bipedal gait likely also impacts disease development. Without a full understanding of the differences between animal models and human disease, the translational potential of these models is weakened. There is a need for an improved understanding of the differences, not just the similarities, between animal models and human disease.

At present, our understanding of OA pathogenesis is heavily dominated by findings from studies in injury-induced OA models. While these studies have provided crucial insight into disease mechanisms, the use of an array of animal models to further investigate the different disease mechanisms and potential drug targets is now critical for progressing OA research given the complexity and heterogeneity of disease presentation in patients. It is particularly timely to re-examine Wnt signalling involvement in OA given that heterogeneity in this pathway has been observed in humans and the emerging evidence that Wnt signalling activity differs between injury-induced mouse models and the STR/ort mouse. Taken together, data from human studies and mouse models indicates that Wnt signalling involvement in OA pathogenesis may differ depending on the causes of OA. Understanding this difference is therefore of critical importance. The implications of species differences in the pathways involved in OA also needs to be better understood. Here, we highlight that differences in TGF-β signalling between mice and humans may mean that the relative importance of TGF signalling to OA pathogenesis differs in humans compared to mouse models. Wnt and TGF signalling are not the only pathways involved in OA pathogenesis. There is a clear need to carefully evaluate the differences in the activity of other OA-relevant pathways between different animal models, as well as between different patients with OA, in order to build an accurate understanding of the mechanisms involved in OA pathogenesis.

## Data Availability

Not applicable.

## Abbreviations

ACLT: Anterior cruciate ligament transection
AXIN 2: Axis inhibition protein 2
BCP: Basic calcium phosphate
BML: Bone marrow lesions
BMP: Bone morphogenic protein
CaMKII: Calcium/calmodulin kinase II
CPP: Calcium pyrophosphate dihydrate
DMM: Destabilised medial meniscus
FLS: Fibroblast-like synoviocytes
FZD: Frizzled
JNK: Jun kinase
MIA: Monosodium iodoacetate
MRI: Magnetic resonance imaging
OA: Osteoarthritis
PCP: Planar cell polarity
PTOA: Post-traumatic
ROR: RAR-related orphan receptor
RYK: Receptor tyrosine kinase related
SMAD: Mothers against decapentaplegic homologue
TGF: Transforming growth factor
WISP 1: WNT1-inducible signalling pathway protein 1
WNT: Wingless-type
